# Effects of virtual reality-based planar motion exercises on upper extremity function, range of motion, and health-related quality of life: a multicenter, single-blinded, randomized, controlled pilot study

**DOI:** 10.1186/s12984-019-0595-8

**Published:** 2019-10-24

**Authors:** Mina Park, Myoung-Hwan Ko, Sang-Wook Oh, Ji-Yeong Lee, Yeajin Ham, Hyoseok Yi, Younggeun Choi, Dokyeong Ha, Joon-Ho Shin

**Affiliations:** 1 Department of Rehabilitation Medicine, National Rehabilitation Center, Ministry of Health and Welfare, 58, Samgaksan-ro, Gangbuk-gu, Seoul, Republic of Korea; 2 Department of Physical Medicine and Rehabilitation, Chonbuk National University Medical School, Jeonju, South Korea; 3 Research Institute of Clinical Medicine of Chonbuk National University, Biomedical Research Institute of Chonbuk National University Hospital, Jeonju, South Korea; 4 Neofect, Yong-in, South Korea; 5 Department of Applied Computer Engineering, Dankook University, Yongin, South Korea

**Keywords:** Virtual reality, Upper extremity, Range of motion, Quality of life, Rehabilitation, Stroke

## Abstract

**Background:**

Virtual reality (VR)-based rehabilitation is considered a beneficial therapeutic option for stroke rehabilitation. This pilot study assessed the clinical feasibility of a newly developed VR-based planar motion exercise apparatus (Rapael Smart Board™ [SB]; Neofect Inc., Yong-in, Korea) for the upper extremities as an intervention and assessment tool.

**Methods:**

This single-blinded, randomized, controlled trial included 26 stroke survivors. Patients were randomized to the intervention group (SB group) or control (CON) group. During one session, patients in the SB group completed 30 min of intervention using the SB and an additional 30 min of standard occupational therapy; however, those in the CON group completed the same amount of conventional occupational therapy. The primary outcome was the change in the Fugl–Meyer assessment (FMA) score, and the secondary outcomes were changes in the Wolf motor function test (WMFT) score, active range of motion (AROM) of the proximal upper extremities, modified Barthel index (MBI), and Stroke Impact Scale (SIS) score. A within-group analysis was performed using the Wilcoxon signed-rank test, and a between-group analysis was performed using a repeated measures analysis of covariance. Additionally, correlations between SB assessment data and clinical scale scores were analyzed by repeated measures correlation. Assessments were performed three times (baseline, immediately after intervention, and 1 month after intervention).

**Results:**

All functional outcome measures (FMA, WMFT, and MBI) showed significant improvements (*p* < 0.05) in the SB and CON groups. AROM showed greater improvements in the SB group, especially regarding shoulder abduction and internal rotation. There was a significant effect of time × group interactions for the SIS overall score (*p* = 0.038). Some parameters of the SB assessment, such as the explored area ratio, mean reaching distance, and smoothness, were significantly associated with clinical upper limb functional measurements with moderate correlation coefficients.

**Conclusions:**

The SB was available for improving upper limb function and health-related quality of life and useful for assessing upper limb ability in stroke survivors.

**Trial registration:**

The study was registered with the clinical research information service (CRIS) (KCT0003783, registered 15 April 2019; retrospectively registered).

## Background

Virtual reality (VR)-based rehabilitation is being increasingly used for post-stroke rehabilitation [1]. A recent systematic review mentioned that VR is an emerging treatment option for upper limb rehabilitation among stroke patients [2]. The benefits of VR include real-time feedback, easy adaptability, and the provision of safe environments that mimic the real world [3, 4]. The gaming property of VR allows patients to experience fun, active participation, positive emotions, and engagement [5, 6]. Therefore, rehabilitation with VR enables more intense and repetitive training, which is important for rehabilitation and the promotion of neural plasticity [7].

VR systems commonly used in the entertainment industry, such as Wii and Kinect, could be used for rehabilitation. However, these game-like systems are only applicable to patients with muscle strength above a certain value, thus limiting their use by more affected patients. Therefore, adjunct therapies, such as functional electrical stimulation and robotics, have been combined with these systems [8–11]. However, those adjunct therapies are costly and require continuous monitoring by healthcare professionals because of safety concerns [12, 13]. Therefore, their use is restricted to clinical settings, and they are not actively used for telerehabilitation or home-based rehabilitation. A non-motorized or non-assisted device is required for more active use of VR for rehabilitation.

We developed the Rapael Smart Board™ (SB; Neofect Inc., Yong-in, Korea), which is a VR-based rehabilitation device incorporating planar motion exercise that does not require additional gravity compensation. This two-dimensional planar movement with full gravitational support, which lessens the need for antigravity muscle facilitation, allows for much easier participation than three-dimensional movement under gravity. Additionally, it is known to be safe and easy to learn, and it has been shown to improve motor ability with less aggravation of shoulder pain and spasticity; therefore, it is useful to patients with reduced motor ability [14]. Planar motion exercises provoke less maladaptive compensatory movements. Additionally, the nearly zero friction of the linear guides enable a wide range of repetitive active range of motion (AROM) exercises. Furthermore, the SB adopted Rapael Clinic software that was originally developed for patients with disabilities and has proven efficacy for stroke rehabilitation [9, 15]. Therefore, the SB, which has multiple advantages because of its hardware and software, might be beneficial for the functional improvement of the upper extremities. Moreover, the SB could have a role as an assessment tool because VR has been reported to be useful for objective kinematic measurements of the upper extremities [16].

The present pilot study aimed to assess the availability of this newly developed VR-based rehabilitation device incorporating planar exercises for the upper extremities as an intervention and assessment tool among stroke patients in the chronic phase of recovery. To assess the availability in terms of clinical effectiveness, we compared the effects of an intervention involving the SB and that involving dose-matched occupational therapy (OT) on upper extremity function and health-related quality of life (HRQoL). We also investigated the correlations between kinematic data from the SB and data from clinical scales regarding upper extremity function.

## Methods

### Study design

This study was a multicenter, single-blinded, randomized, controlled trial that included 26 stroke patients from one rehabilitation hospital and one tertiary university hospital. It was conducted between May 2017 and May 2018. Patients were randomly allocated to the SB intervention (SB) group or conventional intervention (CON) group in a 1:1 ratio using a computer-generated randomization sequence. The assigned group was determined by the consecutive opening of sealed opaque envelopes that contained the name of the group; these envelopes were placed in a plastic container. Randomization was performed centrally, and group allocation was sequentially communicated to the sites via text message.

All patients participated in 20 sets of sessions, 5 days per week, over the course of 4 weeks, which is the usual amount and frequency of treatment at the site where this study was performed. During one session, patients in the SB group completed 30 min of intervention using the SB, whereas patients in the CON group completed the same amount of conventional OT in a research intervention room. During the entire intervention session, occupational therapists were present to assist patients in groups with verbal or physical instructions if necessary. Patients from both groups received an additional 30 min of regular OT in a clinical intervention room.

Outcome measures were performed by experienced occupational therapists (more than 5 years of experience performing stroke rehabilitation including measures and interventions) who were blinded to group allocation. Evaluations were conducted three times (before intervention [T0], immediately after intervention [T1], and 1 month after intervention [T2]).

### Participants

The inclusion criteria were as follows: diagnosis of hemispheric stroke resulting in unilateral upper extremity deficits at least 3 months previously; first-ever ischemic or hemorrhagic stroke; ability to understand instructions, which was confirmed by a score ≥ 25 on the Korean version of the Mini-Mental State Examination; a Medical Research Council (MRC) scale score of 2 or 3 for the strength of the affected upper extremity; and a Modified Ashworth Scale (MAS) score < 2 for spasticity.

The exclusion criteria were as follows: multiple or bilateral brain lesions; age younger than 19 years; any neurological or psychological disorder other than stroke; any severe medical condition; and predisposed to severe pain in the upper extremities that could impede participation in the intervention. A researcher screened inpatient medical records and enrolled participants who were fit for the indication. All participants provided written consent before enrollment in this study, and the study was approved by the institutional review boards of both intervention sites before initiation. The study was registered at the clinical research information service (CRIS; KCT0003783).

### Intervention

#### Smart board intervention

**Fig. 1:**
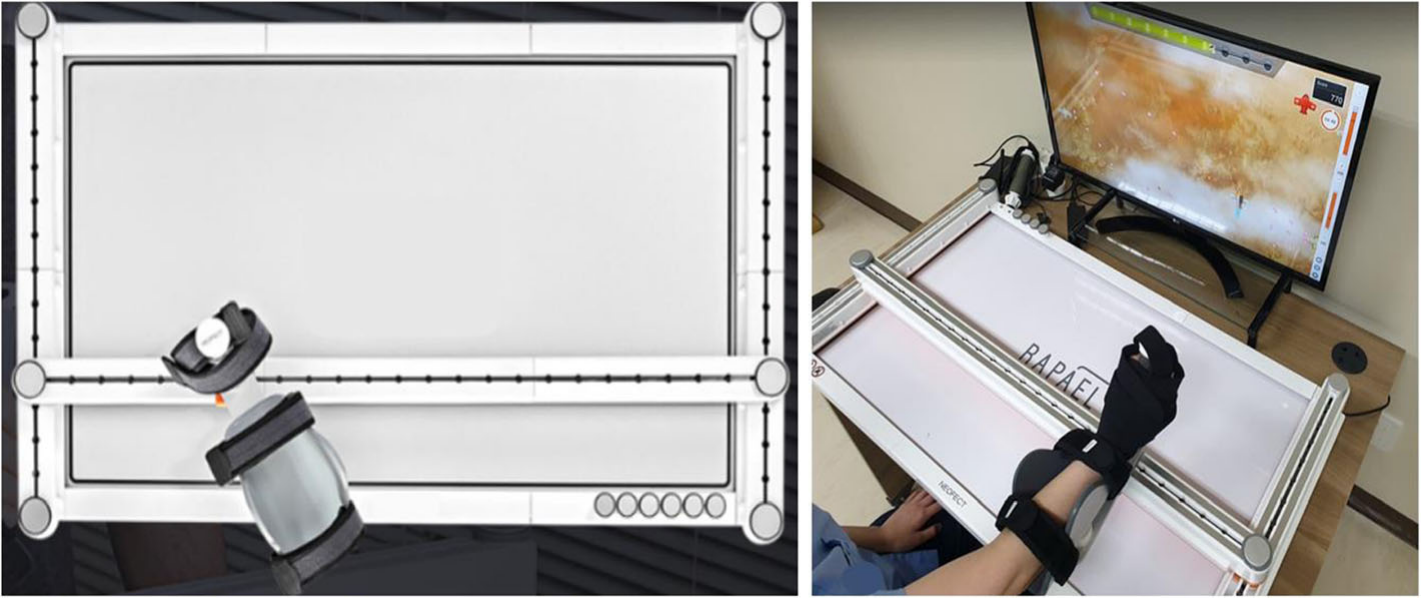
Hardware of the Smart Board. The board and forearm-supported controller. Three linear guides with an H-shape configuration enable two-dimensional planar motion of the handlebar, which is attached to the horizontal linear guide

The SB was developed for patients with impaired upper extremity function. It is focused on proximal upper extremity rehabilitation, including AROM and coordination exercises across multiple joints. It consists of a 104.3-cm × 63.0-cm board with a two-dimensional moving forearm-supported controller, android PC, and display. Three linear guides with an H-shape configuration enable two-dimensional planar motion of the handlebar, which is attached to the horizontal linear guide (Fig. 1). The linear guides have nearly zero friction; therefore, the handlebar moves freely with high precision. Infrared sensors that detect the position of the handlebar continuously are located under the linear guides. Detachable stoppers can be used to limit the maximum moving range of the handlebar and allow one-dimensional movement training. An ergonomically designed armrest allows participants to easily use the SB. A participant is required to hold and move the handle according to the directions provided by Rapael Clinic software. Upper limb movement is visualized as real-time feedback on the system monitor that is linked to the software. Additionally, a printable progress report is presented with training history when the training session is completed. All data are registered in the system individually and are utilized for personalized intervention. The SB assigns a specific gamified intervention among 17 training programs and adjusts the difficulty level to promote active participation in the intervention. The difficulty level of the SB intervention is determined based on the initial assessment before training on the day of participation and the medical history; the specific algorithm for adjusting the level of difficulty has been described previously [15, 17].

#### Smart board assessment program

**Fig. 2:**
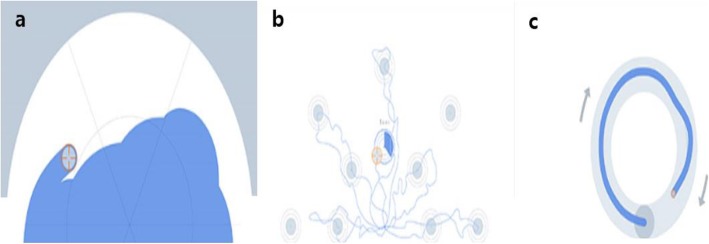
The three different kinematic assessment programs of the Smart Board. The images show the tasks of **a** free exploration, **b** point-to-point reaching, and **c** circle-drawing. The round cursor indicates the movement of the forearm-supported controller

The SB has the following three different kinematic assessment programs: free exploration, point-to-point reaching, and circle-drawing (Fig. 2). During the free exploration task, the participant is required to fill a large half-circle as suggested on the system screen by sweeping over the area as much as possible on the board within a given time, thus reflecting upper extremity AROM and visuomotor mapping ability. During the point-to-point reaching task, a participant is required to move the handle from a centrally located target to 10 targets with five different angles at two different distances, reflecting the ability to perform forward reaching to a given target point. During the circle-drawing task, the participant is required to draw a circle (once clockwise and once counterclockwise), thus reflecting motor coordination. Each assessment program has a time limit of 60 s, and it is automatically terminated even if the task is not completed within that time. Each assessment program has three types of SB kinematic data that are referred to as SB parameters (Table 1).

**Table 1:** Descriptions of Smart Board evaluation tasks and parameters

Task	Parameter	Description
Free exploration	Explored area ratio (%)	The ratio of (a) the area the participant covers to (b) the area required to be filled
	Reaching distance (cm)	The mean reaching distance in all directions
	Completion time (s)	The time required to finish the test
Point-to-point reaching	Target error (cm)	The linear deviation between (a) the target and (b) the point where the speed drops to below 5% of the maximum speed
	Movement time (s)	The mean time to reach the target
	Smoothness	The normalized jerk by integrating the absolute jerk value over all routes
Circle drawing	Shape closeness	The measure of closeness between (a) the target shape (circle) and (b) the shape drawn by the participant
	Roundness	The measure of how closely the shape drawn by the participant approaches that of a mathematically perfect circle
	Completion time (s)	The time required to draw the circle

#### Conventional intervention

The CON intervention focused on the restoration of AROM and coordination of the upper extremities such as AROM exercises for the affected upper extremity, a graded range of motion arc, figure-of-eight tracing using a hand skate, and cone stacking. The CON intervention differs with SB intervention in that this includes some activities embedding vertical plane motion requiring anti-gravity muscle activation instead of planar motion-based activities only. Functional exercise was not incorporated in the CON intervention. Trained occupational therapists modified the type and difficulty level of the intervention according to the participant’s current status. Feedback was provided verbally by the therapists, and physical assistance was provided according to the participant’s status. The SB intervention and CON intervention were conducted in a separate research room for only one participant at a time.

### Outcome measures

We assessed baseline characteristics, including age, sex, dominant hand, stroke type, stroke duration, affected body side, MRC scale scores for the shoulder flexor/extensor and elbow flexor/extensor muscle strength, and upper extremity Fugl–Meyer Assessment (FMA-UE), which is a stroke-specific, performance-based motor impairment scale.

#### Primary outcome

The primary outcome was the change in the FMA-UE score; a higher score indicated lower motor impairment [18]. We used the following three FMA-UE subscales that were relevant to our intervention: FMA-total (33 items; score range, 0–66); FMA-proximal (shoulder, elbow, and forearm; 18 items; score range, 0–36); and FMA-coordination (five repetitions of the finger-to-nose test in rapid succession; three items; score range, 0–6).

#### Secondary outcomes

The secondary outcomes were changes in the Wolf motor function test (WMFT) score, shoulder AROM, Stroke Impact Scale (SIS) score, and modified Barthel index (MBI). The WMFT, which is a quantifiable measurement of upper extremity activity and participation with timed and functional tasks, consists of 17 items, including 15 functional tasks and 2 strength tasks [19]. We used the sum of the functional ability scale scores for items rated using a 6-point scale (WMFT-FAS; higher scores indicate better motor function) and the total amount of time for each item (WMFT-time; shorter time indicates better performance). The AROM of the shoulder joint, including flexion, abduction, adduction, internal rotation, and external rotation, was measured using a goniometer.

The SIS is a stroke-specificself-reported assessment tool for measuring HRQoL. We used the Korean version of the SIS (version 3.0), which contains eight domains scored using a five-point Likert scale (score range, 0–100); higher scores indicate better patient-perspective health status [20, 21]. The SIS was designed to assess multi-dimensional stroke outcomes, including strength, hand function, mobility, activities of daily living (ADLs)/instrumental activities of daily living (IADLs), memory and thinking, communication, emotion, and social participation. Additionally, there is an extra question about stroke recovery that asks participants to indicate how much they feel that they have recovered from stroke (score range, 0–100). The total SIS score was calculated and marked as the overall score. The MBI, which contains 10 items and has a score range from 0 to 100, was used to assess activity and participation [22].

### Statistical analysis

The Mann–Whitney *U* test and Fisher’s exact test were used for baseline comparisons of continuous and categorical variables, respectively, of the study groups. The outcome measures were analyzed using two different approaches. First, within-group changes in the outcome measures were analyzed using the Wilcoxon signed rank test. Second, analyses of the main effects of group (SB and CON), time (T0, T1, and T2), and time × group interactions were conducted by a repeated measures analysis of covariance (ANCOVA) after setting the covariates as stroke duration and baseline FMA score, which affects recovery after stroke. The Greenhouse–Geisser procedure was applied when the sphericity assumption was violated. Statistical analyses of the intervention were performed using SPSS software (version 20.0; IBM Corp., Armonk, NY).

Correlations between repeatedly measured SB parameters and clinical scales, such as FMA-UE, WMFT, and shoulder AROM, were analyzed with repeated measures correlation (rmcorr) using R 3.5.1 (http://www.r-project.org) and R package rmcorr to determine the common linear relationship for paired repeated measures data [23]; *p* < 0.05 was considered statistically significant.

## Results

**Fig. 3:**
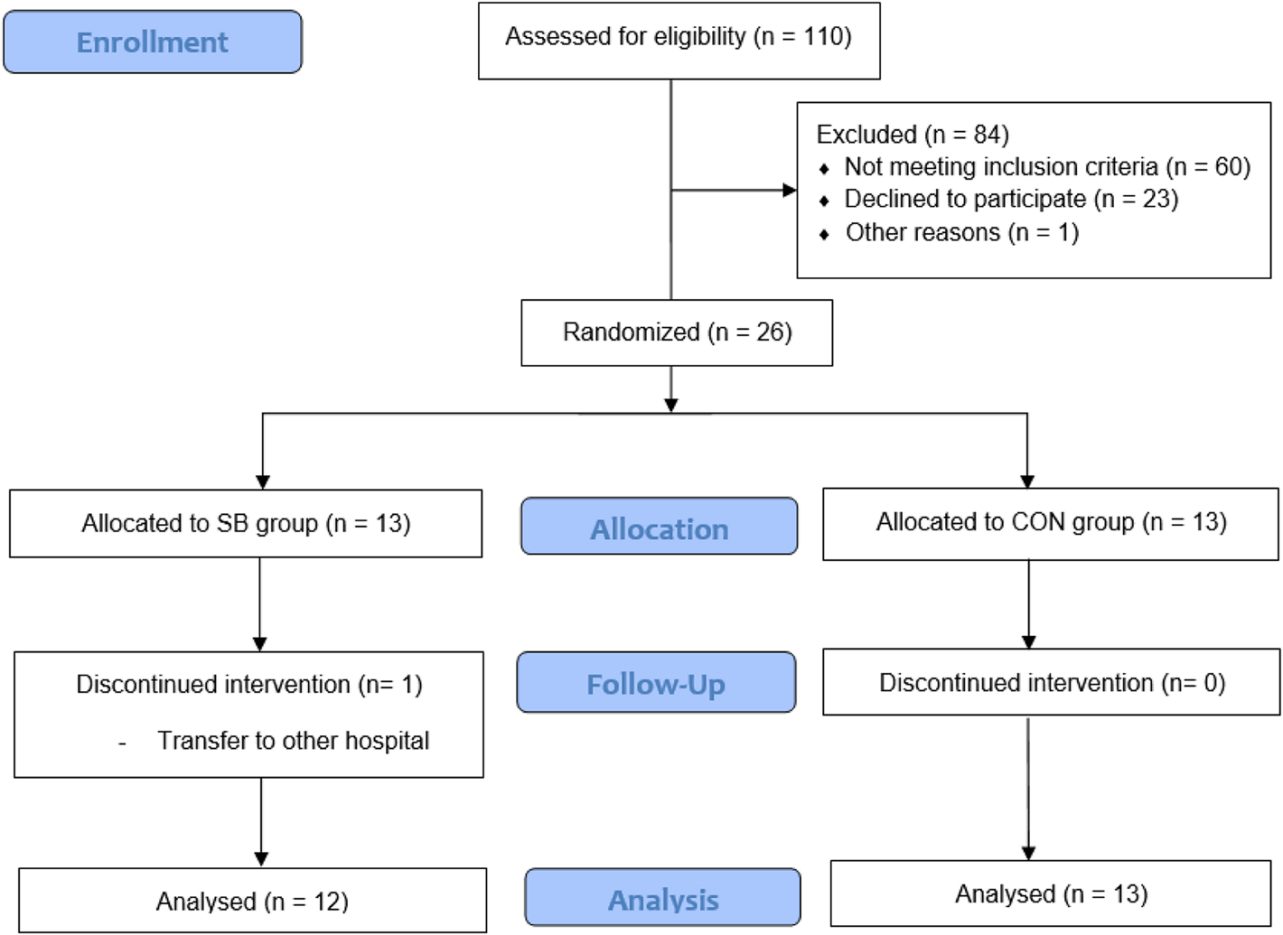
Study flowchart of participants

Of the 26 patients enrolled in the present study, 13 were allocated to the SB group and 13 were allocated to the CON group (Fig. 3). One patient was transferred to another hospital and dropped out of the SB group; therefore, this patient was excluded from the dataset. A total of 25 patients (12 in the SB group and 13 in the CON group) completed the 4-week intervention and were included in the final analysis. No adverse events were observed in either group during the study, which confirmed the safety of the SB intervention. There were no significant differences in baseline characteristics between the two groups (Table 2).

**Table 2:** Demographic and clinical characteristics of the participants

Characteristics	SB group (*n* = 12)	CON group (*n* = 13)	*P*-value
Demographic characteristics			
Age, years	53.5 ± 13.0	51.5 ± 16.7	0.397 ^a^
Sex, male	7 (53.8)	8 (61.5)	0.888 ^b^
Dominant hand, right	13 (100)	10 (76.9)	0.220 ^b^
Stroke characteristics			
Ischemia	5 (38.5)	8 (61.5)	0.813 ^b^
Time from stroke, days	982.3 ± 1473.3	533.5 ± 635.3	0.331 ^a^
Clinical characteristics			
MRC scale shoulder flexor	2.0 ± 0.0	2.2 ± 0.6	0.511 ^a^
MRC scale shoulder extensor	2.5 ± 0.5	2.6 ± 0.9	0.287 ^a^
MRC scale elbow flexor	1.8 ± 0.4	2.2 ± 0.8	0.920 ^a^
MRC scale elbow extensor	2.0 ± 0.7	2.2 ± 0.9	0.579 ^a^
FMA-total score	16.8 ± 7.3	19.9 ± 9.9	0.448 ^a^

Data are presented as mean ± standard deviation or number (%)

*SB* Smart Board intervention, *CON* Conventional intervention, *MRC* Medical Research Council, *FMA* Fugl–Meyer assessment

^a^ Mann–Whitney *U* test, ^b^ Fisher’s exact test

### Primary outcomes

Changes in the FMA-UE scores of the SB and CON groups are presented in Table 3. At the end of the intervention, both groups showed statistically significant improvements in the FMA-total and proximal scores at both T1 and T2, but not the FMA-coordination scores. Repeated measures ANCOVA showed no significant time × group interactions for all FMA-UE subscales. For stroke patients, the minimal clinical detectable change (MCID) was 9 or 10 for FMA-upper extremity [24, 25]. No FMA scores exceeded the MCID in both groups.

**Table 3:** Changes in FMA, WMFT, AROM, and MBI scores and repeated measures ANCOVA outcomes

	SB group (*n* = 12)							CON group (*n* = 13)							RM ANCOVA	
	T0	T1	Change (T1-T0)	*p* -value	T2	Change (T2-T0)	*p*-value	T0	T1	Change (T1-T0)	*p*-value	T2	Change (T2-T0)	*p*-value	F	*p*-value
FMA-total	17.1 ± 7.5	19.0 ± 7.5	1.9 ± 2.8	0.036	19.6 ± 7.5	2.5 ± 3.7	0.014	19.9 ± 9.9	22.0 ± 10.3	2.1 ± 2.7	0.018	25.50 ± 11.8	5.6 ± 8.6	0.012	0.971	0.387
FMA-prox	13.8 ± 3.7	14.9 ± 3.2	1.2 ± 1.8	0.041	15.0 ± 3.0	1.3 ± 2.1	0.041	16.9 ± 6.5	18.4 ± 6.6	1.5 ± 1.8	0.018	19.9 ± 7.1	3.0 ± 4.6	0.035	1.194	0.301
FMA-dist	2.3 ± 3.5	2.9 ± 3.6	0.6 ± 0.9	0.034	3.4 ± 3.7	1.1 ± 1.8	0.024	1.8 ± 2.8	2.2 ± 3.1	0.4 ± 0.9	0.102	4.0 ± 4.2	2.2 ± 3.6	0.018	0.787	0.462
FMA-coor	1.0 ± 1.8	1.3 ± 2.0	0.3 ± 0.9	0.180	1.3 ± 2.1	0.3 ± 0.9	0.180	1.4 ± 1.8	1.5 ± 1.9	0.1 ± 0.6	0.655	1.7 ± 1.9	0.3 ± 1.2	0.414	0.649	0.528
WMFT-FAS	15.0 ± 10.4	19.3 ± 11.1	4.3 ± 5.9	0.012	20.3 ± 11.0	5.3 ± 6.6	0.008	19.6 ± 14.0	24.4 ± 12.9	4.8 ± 5.9	0.003	29.7 ± 15.1	10.1 ± 10.1	0.001	1.451	0.246
WMFT-time (log)	3.1 ± 0.1	3.01 ± 0.1	−0.1 ± 0.1	0.002	3.0 ± 0.1	−0.1 ± 0.1	0.026	3.0 ± 0.2	3.0 ± 0.2	−0.0 ± 0.1	0.016	2.9 ± 0.2	−0.1 ± 0.1	0.023	0.425	0.577
AROM-Sf	68.8 ± 39.9	88.3 ± 45.7	19.6 ± 11.4	0.004	90.0 ± 44.5	21.3 ± 17.1	0.011	85.8 ± 45.4	95.8 ± 44.9	10.0 ± 10.8	0.016	107.3 ± 49.4	21.5 ± 27.9	0.011	0.928	0.373
AROM-Sab	60.8 ± 41.2	75.0 ± 43.8	14.2 ± 16.9	0.007	82.1 ± 53.8	21.3 ± 23.4	0.007	70.8 ± 42.3	77.3 ± 39.9	6.5 ± 8.0	0.026	80.0 ± 39.6	9.2 ± 14.4	0.041	1.516	0.231
AROM-Sad	29.2 ± 10.8	32.9 ± 11.8	3.8 ± 5.7	0.059	33.0 ± 11.8	10.4 ± 12.9	0.059	28.5 ± 14.6	32.3 ± 13.0	3.9 ± 6.5	0.059	33.9 ± 13.3	5.4 ± 6.6	0.020	0.198	0.722
AROM-Sint	35.5 ± 26.0	42.9 ± 22.2	10.4 ± 12.9	0.026	46.3 ± 19.2	13.8 ± 13.0	0.010	36.9 ± 25.2	41.9 ± 24.1	5.0 ± 10.4	0.109	42.7 ± 24.2	5.8 ± 11.2	0.109	2.221	0.137
AROM-Sext	30.0 ± 37.7	33.8 ± 36.4	3.8 ± 5.7	0.059	37.1 ± 34.3	7.1 ± 8.1	0.026	24.2 ± 29.9	30.4 ± 26.7	6.2 ± 10.4	0.066	36.2 ± 29.8	11.9 ± 14.1	0.026	0.335	0.717
AROM-total	221.3 ± 136.2	272.9 ± 134.8	51.7 ± 37.1	0.002	288.3 ± 138.9	67.1 ± 44.3	0.003	246.2 ± 136.8	277.7 ± 123.3	31.5 ± 30.4	0.005	294.4 ± 131.1	53.9 ± 56.5	0.003	0.729	0.454
MBI	69.8 ± 16.4	74.9 ± 16.8	5.1 ± 6.8	0.018	78.4 ± 13.2	8.6 ± 13.1	0.036	70.7 ± 17.9	76.5 ± 15.5	5.9 ± 7.4	0.018	78.9 ± 14.4	8.6 ± 10.2	0.005	0.101	0.848

Data are presented as mean ± standard deviation. The RM ANCOVA covariates were the time from stroke and FMA baseline data

*ANCOVA* Analysis of covariance, *SB* Smart Board intervention, *CON* Conventional intervention, *FMA* Fugl–Meyer assessment (FMA-total, FMA-proximal, FMA-distal, FMA-coordination), *WMFT* Wolf motor function test (WMFT-total, WMFT-functional ability scale, WMFT-time), WMFT-time presented as a log value, *AROM* Active range of motion (*Sf* Shoulder flexion, *Sab* Shoulder abduction, *Sad* Shoulder adduction, *Sint* Shoulder internal rotation, *Sext* Shoulder external rotation), *MBI* Modified Barthel index

### Secondary outcomes

Table 3 presents the changes in secondary outcomes. The WMFT (WMFT-FAS and WMFT-time), MBI, and shoulder AROM of flexion and abduction significantly improved in both groups. Shoulder AROM of internal rotation significantly improved only in the SB group. Additionally, total shoulder AROM and shoulder AROM of flexion, abduction, and internal rotation showed similar or better improvements in the SB group than in the CON group at T1, and these findings continued at T2; however, there was no statistical significance. Repeated measures ANCOVA showed no significant time × group interactions for WMFT, MBI, and shoulder AROM. However, by applying the MCID as which 0.2 to 0.4 for WMFT-FAS, the changes in WMFT-FAS (T1-T0 and T2-T0) showed higher scores than the MCID in both SB and CON groups [26].

Regarding the SIS evaluation, one patient did not complete the assessment due to refusal to participate in the final SIS evaluation. Therefore, the SIS data analysis was performed for 24 patients (12 patients in each of the two groups). Table 4 summarizes the SIS data and their changes during the intervention for the SB and CON groups. The recovery and overall scores showed significant differences between T0 and T1 only in the SB group. The emotion and communication scores showed improvements in the SB group and deterioration in the CON group. Repeated measures ANCOVA showed statistically significant time × group interactions for memory and thinking and the overall SIS score, with larger differences observed in the SB group.

**Table 4:** Changes in SIS scores in the SB and CON groups and repeated measures ANCOVA outcomes

	SB group (*n* = 12)							CON group (*n* = 12)							RM ANCOVA	
	T0	T1	Change (T1-T0)	*p*-value	T2	Change (T2-T0)	*p*-value	T0	T1	Change (T1-T0)	*p*-value	T2	Change (T2-T0)	*p*-value	F	*p*-value
Strength	22.3 ± 14.0	29.4 ± 18.0	7.2 ± 10.0	0.034	27.3 ± 20.0	5.1 ± 12.2	0.153	19.7 ± 11.4	28.3 ± 13.1	8.58 ± 10.7	0.027	27.0 ± 14.3	7.3 ± 12.5	0.065	0.327	0.723
Hand function	0.8 ± 1.9	2.9 ± 5.8	2.1 ± 5.8	0.180	2.9 ± 6.2	2.1 ± 6.6	0.285	5.4 ± 8.6	6.3 ± 12.1	0.8 ± 8.5	0.891	8.3 ± 13.7	2.9 ± 12.1	0.357	0.086	0.858
Mobility	50.7 ± 26.0	53.6 ± 20.0	2.9 ± 15.9	0.386	58.5 ± 18.1	7.8 ± 16.5	0.103	69.9 ± 28.9	68.0 ± 30.3	− 1.9 ± 7.9	0.675	73.7 ± 29.1	3.8 ± 7.0	0.046	1.844	0.171
ADLs/IADLs	59.2 ± 15.6	61.7 ± 14.7	2.5 ± 13.6	0.789	62.9 ± 12.6	3.8 ± 12.5	0.307	64.5 ± 15.5	62.5 ± 15.1	− 2.0 ± 6.1	0.259	70.8 ± 16.3	2.5 ± 6.1	0.085	0.667	0.519
Memory and thinking	70.2 ± 20.8	80.1 ± 15.4	9.9 ± 16.1	0.052	76.7 ± 16.9	6.5 ± 16.9	0.258	86.3 ± 10.0	85.5 ± 13.5	− 0.8 ± 7.3	0.865	88.8 ± 11.2	6.3 ± 10.1	0.172	4.484	0.017
Communication	83.8 ± 20.1	90.4 ± 16.8	6.6 ± 18.8	0.123	89.9 ± 19.1	6.1 ± 18.9	0.123	95.2 ± 5.5	90.7 ± 10.4	−4.5 ± 6.2	0.034	92.8 ± 9.5	−2.3 ± 5.4	0.167	4.009	0.051
Emotion	65.1 ± 17.9	70.8 ± 15.1	5.7 ± 15.5	0.182	70.8 ± 18.6	5.7 ± 12.5	0.212	80.5 ± 13.7	79.0 ± 15.8	−1.5 ± 5.5	0.475	80.1 ± 15.3	− 0.4 ± 8.9	0.959	1.901	0.163
Social participation	36.2 ± 20.6	42.8 ± 23.2	6.6 ± 10.1	0.050	41.3 ± 22.5	5.2 ± 9.9	0.096	39.3 ± 12.3	41.5 ± 15.7	2.2 ± 14.5	0.574	41.7 ± 11.1	2.3 ± 9.5	0.444	0.144	0.867
Recovery	31.7 ± 13.5	43.3 ± 13.9	11.7 ± 13.2	0.004	45.0 ± 13.7	13.3 ± 13.9	0.004	47.5 ± 17.1	49.6 ± 15.3	2.1 ± 8.1	0.493	57.5 ± 17.0	10.0 ± 10.2	0.011	2.673	0.081
Overall	388.2 ± 93.4	431.6 ± 58.4	43.4 ± 56.1	0.033	430.3 ± 70.8	42.2 ± 60.5	0.041	460.8 ± 72.0	461.7 ± 85.3	0.8 ± 24.3	0.824	483.3 ± 89.4	22.4 ± 30.2	0.018	3.559	0.038

Data are presented as mean ± standard deviation. The RM ANCOVA covariates were the time from stroke and FMA baseline data

*SIS* Stroke impact scale, *ANCOVA* Analysis of covariance, *SB* Smart Board intervention, *CON* Conventional intervention, *ADLs/IADLs* Activities of daily living/instrumental activities of daily living

### SB assessment

**Fig. 4:**
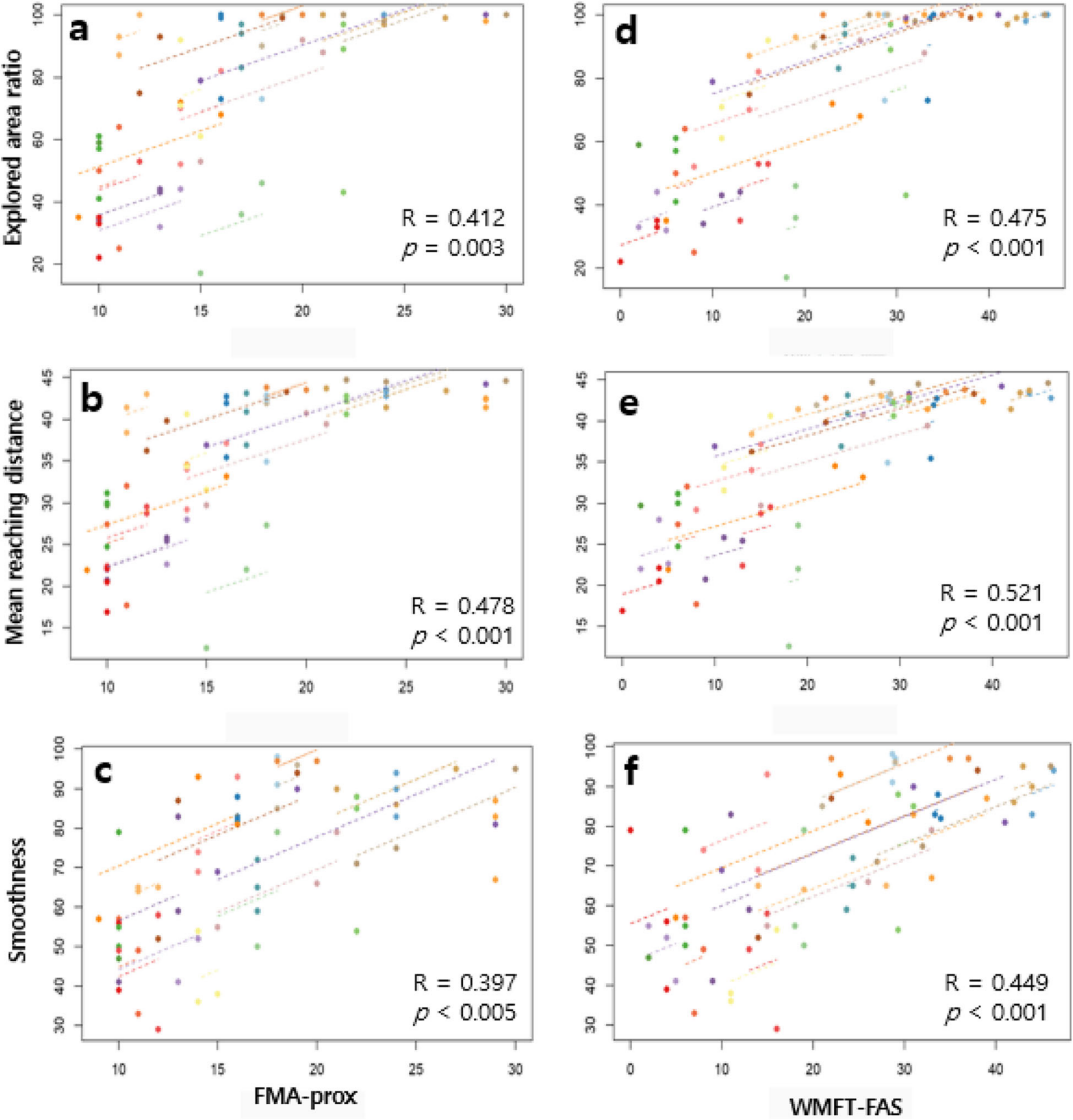
Repeated correlation graphs. Correlations between repeatedly measured clinical scales (**a**-**c** FMA-prox; **d**-**f** WMFT-FAS) and Smart Board parameters (**a** and **d** explored area ratio; **b** and **e** mean reaching distance; **c** and **f** smoothness) were analyzed by repeated measures correlation (rmcorr). R indicates the correlation coefficient. FMA, Fugl–Meyer assessment; WMFT, Wolf motor function test

A repeated measures correlation analysis showed that changes in the three SB assessment aspects (explored area ratio [EAR], mean reaching distance [MRD], and smoothness [Sm]) were significantly correlated with changes in clinical scale scores such as FMA-total, FMA-prox, WMFT-FAS, WMFT-time, and shoulder AROM. In particular, AROM total score and MRD showed the highest correlation coefficients (*R* = 0.592; *p* < 0.001). Figure 4 demonstrates the FMA-prox and WMFT-FAS graphs that showed high correlation with the three aforementioned parameters (EAR, MRD, and Sm). However, parameters such as target error (TE) and roundness (Ro) showed no significant correlation with any clinical parameters we examined. Generally, the SB parameters showed higher correlation coefficients with WMFT scales than with FMA scales. The FMA-coordination scale was not correlated with any SB parameter (Table 5).

**Table 5:** Correlation analysis of SB parameters and clinical scales

Task	Free exploration			Point-to-point reaching			Circle drawing		
Parameter	EAR	MRD	ECT	TE	MT	Sm	SC	Ro	CCT
FMA-total	0.360*	0.414**	−0.210	− 0.063	− 0.112	0.342*	0.185	0.089	−0.232
FMA-prox	0.412**	0.478**	−0.146	−0.069	− 0.152	0.397**	0.228	0.107	−0.240
FMA-dist	0.311*	0.348*	−0.267	−0.055	− 0.060	0.267	0.109	0.036	−0.223
FMA-coor	0.172	0.138	−0.134	−0.006	− 0.028	0.069	0.120	0.019	−0.083
WMFT-FAS	0.475**	0.521**	−0.297*	−0.245	− 0.228	0.449**	0.219	0.045	−0.287*
WMFT-time	−0.400**	−0.441**	0.139	0.277	0.363*	−0.344*	−0.261	− 0.041	0.303*
AROM-Sf	0.577**	0.635**	−0.240	−0.203	− 0.252	0.424**	0.376**	−0.009	− 0.084
AROM-Se	−0.081	− 0.025	−0.133	− 0.226	−0.223	0.045	0.090	−0.046	0.109
AROM-Sab	0.337*	0.389**	−0.380**	−0.052	− 0.157	0.213	0.261	−0.009	− 0.183
AROM-Sad	0.465**	0.470**	< −0.001	−0.041	− 0.395**	0.378**	0.270	0.007	0.001
AROM-Sint	0.505**	0.503**	−0.199	−0.015	− 0.441**	0.324*	0.238	0.063	− 0.309*
AROM-Sext	0.441**	0.497**	−0.299*	−0.173	− 0.050	0.072	0.141	0.098	−0.178
AROM-total	0.529**	0.592**	−0.337*	−0.186	− 0.344*	0.364*	0.353*	0.009	−0.155

*EAR* Explored area ratio (%), *MRD* Mean reaching distance (cm), *ECT* Exploration completion time (s), *TE* Target error (cm), *MT* Movement time (s), *Sm* Smoothness, *SC* Shape closeness, *Ro* Roundness, *CCT* Circle completion time (s), *FMA* Fugl–Meyer assessment (FMA-total, FMA-proximal, FMA-distal, FMA-coordination), *WMFT* Wolf motor function test (WMFT-total, WMFT-functional ability scale, WMFT-time), *AROM* Active range of motion (*Sf* Shoulder flexion, *Sab* Shoulder abduction, *Sad* Shoulder adduction, *Sint* Shoulder internal rotation, *Sext* Shoulder external rotation)

The number means correlation coefficient (R) and *P*-values are presented as *p < 0.05, **p < 0.01

## Discussion

The SB, which is a newly developed VR-based planar exercise apparatus, was available for stroke rehabilitation in terms of clinical effectiveness. We found that the SB intervention with conventional OT improved upper extremity function, and that the effects on the SB group were not different from those on the CON group, without any adverse events. Notably, greater improvement was observed in shoulder AROM in the SB group than in the CON group, although there was a lack of statistical significance. This was meaningful because upper limb AROM is closely related to upper extremity function among stroke survivors, and shoulder AROM was found to explain 65% of the variance in upper extremity function [27, 28]. The largest difference between the SB and CON groups was noted for shoulder abduction, which is known to be closely related to upper limb functional recovery [29].

The beneficial effects of the SB on AROM might be associated with the hardware characteristics of the SB. Although AROM exercises have been emphasized for stroke rehabilitation, they have not been performed sufficiently because they are difficult to perform alone and the absolute amount of rehabilitation is limited. The SB attempted to overcome these limitations by using easily movable non-motorized equipment in combination with VR. Specifically, the SB was designed to be non-motorized but free from friction so that it would be suitable for repetitive movement without assisted force. Moreover, simple two-dimensional exercise could be intensively practiced using visual cues on the monitor during training. This planar movement training might be appropriate during the preliminary phase of recovery, when motor performance is not sufficient for complex movement training.

Regarding the SIS, the SB with conventional OT showed better results for improving the overall score of HRQoL when compared with the same amount of conventional OT, and it showed positive effects for many domains of the SIS. A recent Cochrane review could not conclude the role of VR with regard to quality of life because of the lack of studies [2]. However, similar to the findings of our previous studies, the present findings support the beneficial effects of VR on HRQoL after stroke rehabilitation [15, 30]. Interestingly, there was a marked difference between SIS-emotion scores of the two study groups, thus reflecting the strength of the gaming properties of the SB. Similarly, a previous study showed that VR with serious games significantly increased the Beck Depression Inventory score [31]. These emotional changes might occur gradually with positive experience, because gaming properties, including immediate and concrete feedback, reappraisal, challenging tasks, and optimal difficulty levels, can provide an enjoyable feeling and a feeling of achievement, which help motivate active participation in the rehabilitation process, thereby leading to better functional outcomes [30, 32–34]. A recent review demonstrated that VR and active video gaming have positive effects on a patient’s motivation and enjoyment [35].

The SB group showed relatively notable improvements in the SIS-ADLs/IADLs domain, despite limited differences in the MBI when compared with the findings of the CON group. Therefore, positive emotional improvements might have contributed to improved subjective perception of ADLs because the SIS reflects the perceived ability of ADLs, whereas the MBI reflects the real ability.

In addition to therapeutic effects, the SB allows for a simple, easily applicable, objective, and quantitative assessment of motor performance. VR has not been commonly used for assessments, although it is popular in the therapy field. Similarly, correlations between VR-based measurements and clinical scales for assessing upper extremity function have not been sufficiently explored. The SB as a new VR-based assessment tool could be used to evaluate upper limb function with simultaneous recordings of kinematic data using an SB-embedded sensor without the need to attach additional sensors to the participant during a short period of 3 min.

The SB parameters significantly matched the clinical scales for upper limb function and their changes. Among the nine SB parameters used, three (EAR, MRD, Sm) were moderately correlated with the FMA and WMFT, suggesting that evaluation with the SB allows for estimation of the upper extremity function across impairment and activity domains. Therefore, the SB assessment might help provide precise and reliable feedback to participants. Fundamentally, evaluation using the SB has the advantage of providing objective measurements of movement performance that could complement conventional clinical assessments.

The SB allows more immediate and frequent feedback to therapists and participants, potentially allowing more individualized rehabilitation. Although the correlation coefficients were not high enough to consider replacing clinically established scales, we obtained moderate correlations that were comparable with robotic measurements, which are more costly than our measurements [36]. Further combinations of multiple metrics could lead to a higher estimation of competence of the SB [37].

SB parameters are activity-relevant and specific. The correlation coefficients for the SB parameters (EAR, MRD, and Sm) and the WMFT were higher than the coefficients for the parameters and the FMA, indicating that the SB parameters reflect activity and participation more than impairment. Correlations with shoulder AROM were greater during the free exploration task than during the other two tasks (point-to-point reaching and circle-drawing tasks) because of the similarity between free exploration and AROM.

In contrast to the positive results of these SB parameters, the time-relevant factors of free exploration and point-to-point reaching (completion time and movement time) showed only limited significant correlations with clinical scales. This might be associated with the floor effects of those parameters because most of the participants did not finish the free exploration and point-to-point reaching tasks within 1 min; therefore, the movement time was nearly the same among the participants. In contrast, the time variables of the circle-drawing task, which most participants could finish within 1 min, showed significant correlations with the WMFT. Therefore, it is necessary to modify the approaches by adjusting the difficulty or altering the calculation method to increase diagnostic validity.

The SB originally targeted both AROM exercises and coordination at the development stage; however, the SB intervention failed to achieve significant improvements in coordination. There is still a lack of evidence indicating that coordination could be improved with rehabilitation, and most improvements occur during the acute phase of stroke because of spontaneous recovery rather than during the chronic phase [38, 39]. Similarly, the SB intervention was incapable of accomplishing notable voluntary control ability associated with coordination. Moreover, we did not find any significant coordination-correlated SB parameters because FMA-coordination, which includes only three items (tremor, dysmetria, and speed), might not be sufficient to reflect coordination compared to more specific outcome measures such as inter-joint movement analysis or sub-movement analysis [40, 41].

The present study had several limitations. First, our results should be carefully interpreted because both the SB and CON groups received 30 min of OT in addition to the primary intervention. Therefore, the effects could not be solely attributed to either intervention. Second, the sample size was small and the number of participants was not calculated with a power analysis because the present study was a preliminary study performed to assess availability, thus limiting the ability to draw a definite conclusion. Therefore, a future study with more participants for comparison of a single intervention is needed. Finally, the SB kinematic data have not been evaluated for their reliability even though the infrared sensor is able to present accurate measurements. Therefore, a reliability test might be needed to utilize the SB kinematic data as a more reliable outcome measurement.

## Conclusion

VR-based rehabilitation incorporating planar motion exercises is available as an intervention and assessment tool for stroke patients. It could be a user-friendly rehabilitation approach for not only upper extremity function and AROM but also HRQoL. Additionally, easily applicable assessments, which are moderately correlated with clinical scales and associated with a short time period, could broaden and support the role of VR in rehabilitation.

## Data Availability

The dataset used in the present study is available from the corresponding author on reasonable request.

## Abbreviations

ADLs/IADLs: Activities of daily living/instrumental activities of daily living
ANCOVA: Analysis of covariance
AROM: Active range of motion
CON: Control
FMA: Fugl–Meyer assessment
HRQoL: Health-related quality of life
MAS: Modified Ashworth Scale
MBI: Modified Barthel index
MRC: Medical Research Council scale
OT: Occupational therapy
rmcorr: Repeated measures correlation
SB group: SB intervention group
SB: Rapael Smart Board™
SIS: Stroke Impact Scale
WMFT: Wolf motor function test
